# Deep generative model for drug design from protein target sequence

**DOI:** 10.1186/s13321-023-00702-2

**Published:** 2023-03-28

**Authors:** Yangyang Chen, Zixu Wang, Lei Wang, Jianmin Wang, Pengyong Li, Dongsheng Cao, Xiangxiang Zeng, Xiucai Ye, Tetsuya Sakurai

**Affiliations:** 1 Department of Computer Science, University of Tsukuba, Tsukuba, 3058577 Japan; 2 College of Computer Science and Electronic Engineering, Hunan University, Changsha, 410082 Hunan People’s Republic of China; 3 Xiangya School of Pharmaceutical Sciences, Central South University, Changsha, 410013 Hunan China; 4 School of Computer Science and Technology, Xidian University, Xian, 710071 China; 5 The Interdisciplinary Graduate Program in Integrative Biotechnology and Translational Medicine, Yonsei University, Incheon, 21983 Republic of Korea; 6 Bioinformatics and Molecular Design Research Center (BMDRC), Incheon, 21983 Republic of Korea

## Abstract

Drug discovery for a protein target is a laborious and costly process. Deep learning (DL) methods have been applied to drug discovery and successfully generated novel molecular structures, and they can substantially reduce development time and costs. However, most of them rely on prior knowledge, either by drawing on the structure and properties of known molecules to generate similar candidate molecules or extracting information on the binding sites of protein pockets to obtain molecules that can bind to them. In this paper, DeepTarget, an end-to-end DL model, was proposed to generate novel molecules solely relying on the amino acid sequence of the target protein to reduce the heavy reliance on prior knowledge. DeepTarget includes three modules: Amino Acid Sequence Embedding (AASE), Structural Feature Inference (SFI), and Molecule Generation (MG). AASE generates embeddings from the amino acid sequence of the target protein. SFI inferences the potential structural features of the synthesized molecule, and MG seeks to construct the eventual molecule. The validity of the generated molecules was demonstrated by a benchmark platform of molecular generation models. The interaction between the generated molecules and the target proteins was also verified on the basis of two metrics, drug–target affinity and molecular docking. The results of the experiments indicated the efficacy of the model for direct molecule generation solely conditioned on amino acid sequence.

**Supplementary Information:**

The online version contains supplementary material available at 10.1186/s13321-023-00702-2.

## Introduction

The chemical space of drugs is estimated to be between 10^23^ and 10^60^ power of molecules. Despite the compound library contains millions of data, easily scalable compound libraries have covered only a tiny fraction of the synthesized, drug-like chemical space [1, 2]. Obtaining ligands (representing drugs or compounds) for proteins is a critical task in drug discovery. Given a specific protein (also called target, such as a protein representing a human or virus), two standard methods could be reemployed to discover a ligand: virtual screening and de novo design. Virtual screening [3] is carried out in two manners: (1) by studying the interaction patterns between target proteins and small molecules and (2) by comparing the structures and pharmacophores of known small molecules to select from many molecules that are reasonable for subsequent testing. De novo design towards target protein directly generates molecules by exploring the explicit rules of known data, in contrast to virtual screening, which is cumbersome and not straightforward.

Molecules with novel structures are constructed by combining existing compound fragments or genetic algorithms normally in the early stages of drug design. Recently, deep neural networks (DNNs)-based models gradually make their mark in a myriad of domains. Accordingly, methods based on deep learning (DL) [4] have a wide range of applications in the field of de-novo design [5, 6] and support variable molecule format, including molecule fingerprints [7], simplified molecular input line entry system (SMILES) [8], molecule graphs [9, 10], and three-dimensional (3D) structures [11]. They try to learn the probability distribution from relevant data, explore latent features, and infer new molecules from the learned data distribution. Furthermore, the generative model combined with reinforcement learning (RL) [12] could optimize the generated molecules [13, 14]. In summary, the application of deep generative networks has made it possible to take a big step forward in drug discovery.

**Fig. 1:**
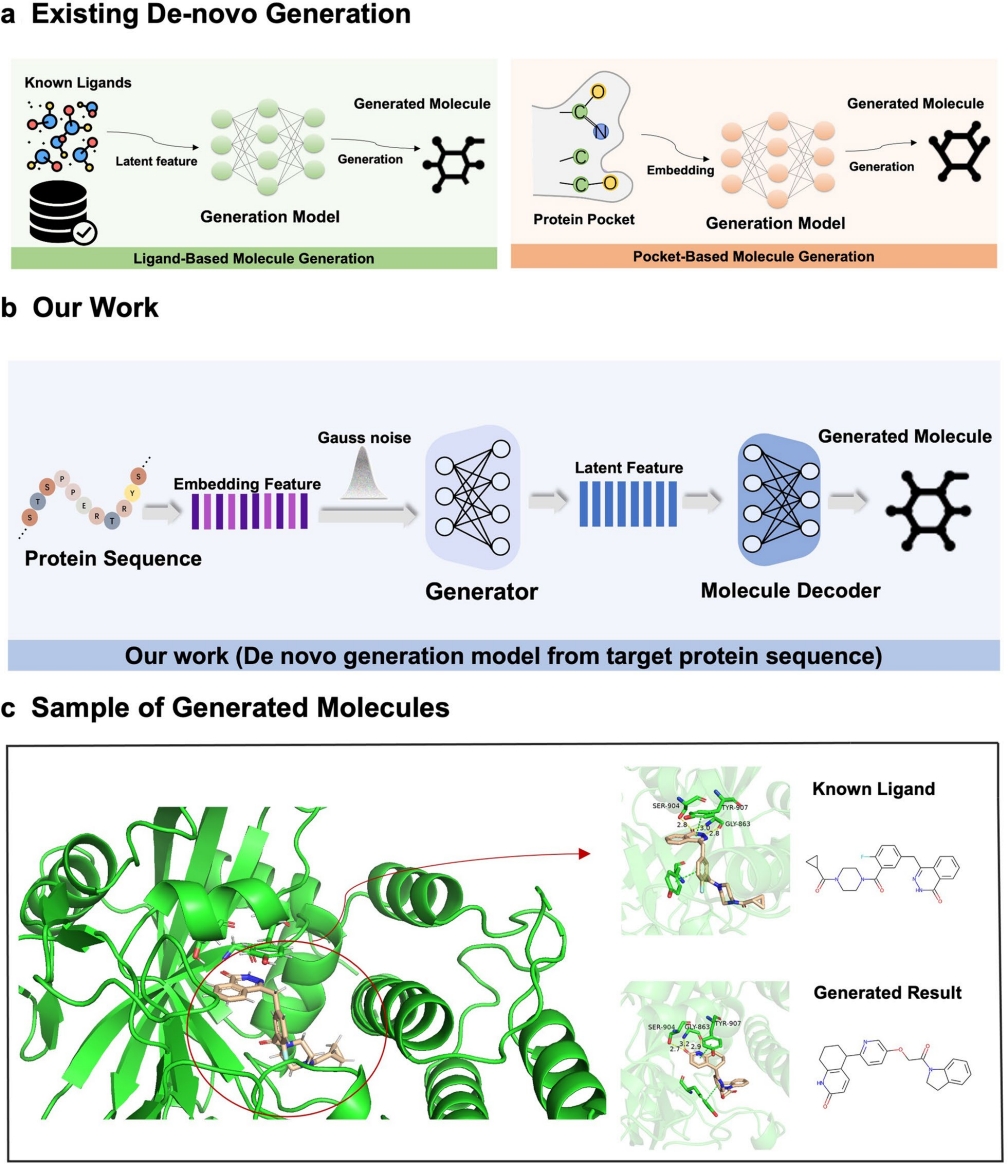
**a** Existing De-novo Generation: Ligand-Based Molecule Generation (LBMG) and Pocket-Based Molecule Generation (PBMG). LBMG methods generate new molecules based on the backbone structure and properties of existing ligands. PBMG methods are conditioned on known bind site information about proteins. **b** Our model only relies on protein sequence to directly generate molecules that interact with targets. **c** Sample with same binding site against target protein

Existing generation methods (Fig. 1a) are divided into ligand-based molecule generation (LBMG) and pocket-based molecule generation (PBMG). LBMG methods generate novel molecules on the basis of the backbone structure and properties of existing ligands. For example, 3DMolGNN [15] refines the molecule (produced by a pre-trained molecule generation network) as close as possible to the known inhibitors of the same family of proteins by transfer learning. It produces small molecules that better bind to the target protein and exhibit desirable drug properties. Merk et al.[16] inputs SMILES strings and fine tunes on a smaller activate drug dataset to force the models to generate focused molecule libraries with the desired activity towards the same target. GENTRL [17] utilizes a generative model combining with combined reinforcement learning (RL), variational inference, and tensor decompositions to design inhibitors of discoidin domain receptor 1 (DDR1). It is pretrained using the ZINC database and then continued with DDR1 and common kinase inhibitors. As for PBMG methods, they are conditioned on known bind site information about proteins. For example, MolAIcal [18] generates 3D structures of molecules from 3D pockets of protein targets, and it is based on the PDBbind database and docking screening by Autodock Vina [19]. Miha et al. [20] trained a generative adversarial model to generate compound structures that are complementary to protein through the obtained structures of protein–ligand complexes. Methods, such as LiGAN and GVAE_SVAE [21, 22], encode protein pockets and choose conditional VAE to generate new molecules that could bind to protein pockets. Similarly, EGCM_cRNN [23] integrates 3D structural information of protein binding pockets into conditional RNN (cRNN) models to control the generation of drug-like molecules.

Although previous works have made great progress in drug generation, they suffer from different problems. LBMG methods could not jump out of the existing chemical space [17, 24], making it hard to generate molecules with very novel structures. Though those methods could generate extremely high similarity results compared with reference molecule, the result they generate is a few new atoms added to the original molecule. They could not be used to search all spaces for bioactive molecules, and they do not contribute substantially to the new drug discovery. In addition, some proteins only have few known ligands. Thus, they cannot supply sufficient data to train a model. PBMG methods require advanced knowledge of the protein, such as 3D structure or pocket information. However, determining the 3D protein structure is extremely expensive, and the 3D structures of many proteins are still uncovered. While AlphaFold [25] provides a computational method that could predict protein structures, the accuracy of the forecasts could not be guaranteed, especially for proteins that have few intra-chain or homotypic contacts. In addition, some proteins have more than one protein pockets. Literature or database research, experimental screening, or software predictions could help predict the active pocket or identify the binding site of ligand, but they are also laborious. In summary, these problems could be a hindrance to ligand design.

In this research, considering the complexity of the previous approach, a model (Fig. 1b) named DeepTarget was proposed for generating potent ligand for protein targets, only based on protein sequences. The method does not need to concern information about protein pockets, nor does it need to be fine-tuned in a specific range of molecular libraries. The protein sequence is simply provided to obtain the corresponding molecule. Generative adversarial networks (GANs) [26, 27] were chosen as the underlying structure of the generative model for this task, combining conditional models with contrastive learning (CL)[28] to further constrain and improve the nature of the generated results. The main contributions of the present work are as follows:

1. The model requires neither prior knowledge of protein binders nor preparation of a library of ligands active against the target, thus allowing molecules generation based on protein amino acid sequence only.
2. An exhaustive series of experiments was carried out on the model. The model performed strong target-based molecule generation capabilities under various metrics.
3. We performed a molecular docking analysis of the generated results on two representative proteins (DRD2 and Parp1) and demonstrated the generated molecules can bind with the target proteins.

## Methods

### Datasets of details

Data were collated from the ChEMBL database [29]. The original dataset contains 1,417,957 drug–protein pairs, combined with unlimited protein types and a positive-to-negative sample ratio of approximately 1:1. The following actions were applied to select records from the original data:

1. All activity values under each target were marked to the median based on the median value, and if the activity value was greater than the median, it was marked as 1 and vice versa.
2. Data with label of 0 were removed, and data with label of 1 were chosen.
3. SMILES was processed, and invalid SMILES molecular formulas were removed using RDKit, including removing salts and stereo-chemistry from the SMILES sequence. Sequence corresponding to inorganic molecules or those that could not be interpreted by RDKit were removed.
4. Proteins with unknown token “X” were removed.
5. Compounds with uncommon atoms were removed.
6. Characters of SMILES containing the isomer information were removed, and the strings containing the following tokens were reserved.
  $$ \{ C,c,N,n,S,s,O,o,F,{\text{Cl}},[nH],{\text{Br}},{\text{1}},{\text{2}},{\text{3}},{\text{4}},{\text{5}},{\text{6}},{\text{7}},{\text{8}},9,\# , = , + , - ,(,),[,]\}  $$
7. Sequence containing the following tags in the protein target were reserved.
  $$\{K,I,F,W,Y,H,A,T,B,G,V,E,P,L,N,Q,S,U,D,R,C,M\}$$

After screening was conducted, the training set consists of 551,223 drug–protein pairs, with 1,970 unique proteins and 333,399 unique SMILES sequences. Among them, a fraction of the protein targets corresponds to less than 10 drug molecules that could interact with each other, while the largest number of targets has 3091 corresponding data.

### Model structure

**Fig. 2:**
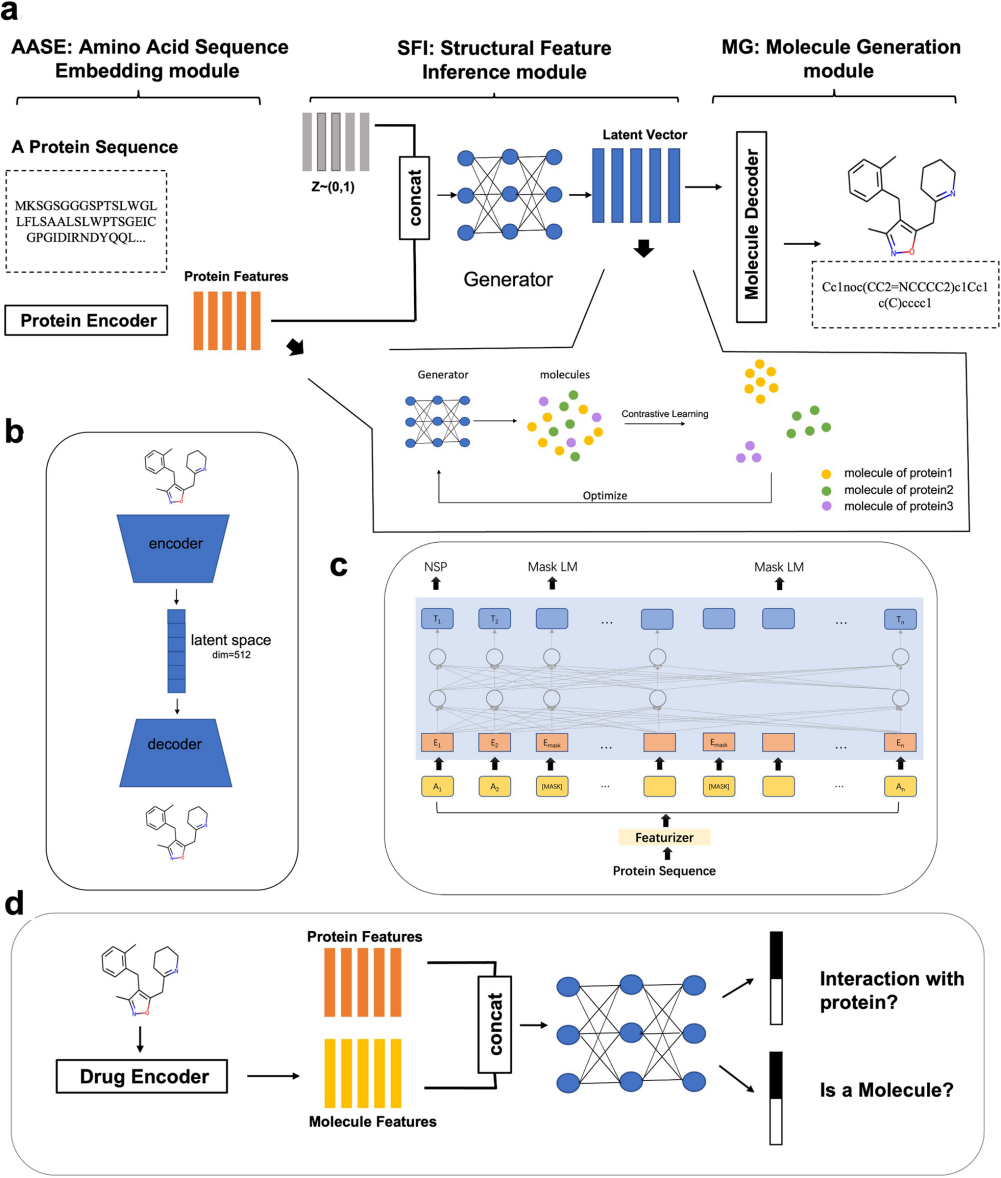
**a** Three modules of DeepTarget: the Amino Acid Sequence Embedding module (AASE), the structural feature inference module (SFI) and the molecule generation module (MG). **b **The autoencoder model, encoder is to embed SMILES to latent feature and decoder is to access SMILES from generated results. **c**The classical Transformer architecture that is proposed to get the features of the protein sequence. **d** The discriminator in SFI, which which works against the generator

As shown in Fig. 2a, DeepTarget consists of three modules: AASE, SFI, and MG. Details of the modules were presented below.

### AASE

This module is constructed by a classical Transformer [30] architecture. It incorporates a 12-layer Transformer with a hidden size of 512 units and eight attention heads. The hyperparameters of the model are approximately the same as those in the Transformer. The model was trained by masked-token prediction (Fig. 2c). First, the protein sequence was encoded by IUPAC Tokenizer [31]. Second, a part of the token was randomly masked in the input sequence (i.e., the original token is replaced with “[MASK]”), and then the output vectors in the masked position were predicted. After the above pre-training, fine-tuning was conducted in contact prediction, sequence classification, or other missions. Lastly, a 756-dimensional feature vector, which was used to represent the features of the protein sequence, could be obtained.

### SFI

Subsequently, a condition generator was constructed by inputting protein embedding feature as condition. The Wasserstein GANs with gradient penalty (WGAN-GP) described in [32] were adopted due to their good performance in generator realistic images/text and model stability. The generation process could be expressed as follows:

$$z=N\sim (\mathrm{0,1})$$

$$c=MLP\left(P\right)$$

$$S=G\left(z,c\right)$$

Initially, the protein feature $P$ embedded in AASE was converted into a 512-dimension common space $c$ by a perception layer. Then, it was concatenated with a random generated noise $z$ that conformed to normal distribution. Finally, the combined vector was inputted into the condition generator and processed by multi-layer neural networks to obtain the final result $S$, which represents the latent feature of the expected molecule.

### MG

The latent feature generated by SFI was inputted into a decoder architecture of an autoencoder model trained in the ChEMBL dataset. LSTM was applied as the internal module in the autoencoder because of its strong capacities of reconstruction and learn molecule distribution [33, 34]. The architecture of the autoencoder [35] is illustrated in Fig. 2b. The autoencoder is able to encode and decode the molecules by pre-training on a large-scale dataset. In the model, the autoencoder's encoder is used to encode known molecules and the GANs in the SFI module learn the encoded feature distribution so that it can generate similar molecular features. Specifically, during the encoding stage, a SMILES string was encoded as one-hot format was inputted into a two-layer bidirectional network with 512 LSTM units per layer, half of which was used in the forward direction and half in the backward direction. Afterwards, all the outputs were concatenated and inputted to a 512-dimension feed-forward layer. The 512-dimension latent vector was assumed to represent the latent feature of the SMILES. During the decoding stage, the latent representation of the molecule was inputted into a four-layer LSTM decoder model that trained under teacher forcing mechanism, generally similar to the encoder. Ultimately, the output of the last network was fed to a feed-forward layer with softmax activation. Thus, the sampling probability of each word could be obtained and the corresponding SMILES could be decoded. In MG, the latent feature for SMILES of the molecule replaced the encoder feature, and it was processed by a trained decoder module to obtain the expected molecule. The parameters of the autoencoder model pre-trained by[36] were directly chosen and were frozen in the MG module for reconstructing. They were trained on extremely large SMILES from the ChEMBL dataset, and good performance was achieved.

## Objective functions

The loss function is made up of two main parts, which come from the generator G and the discriminator D.

During training, the generator G and discriminator D were trained alternately. In detail, the generator G was trained by minimizing the following loss:

$${\mathcal{L}}_{{G}_{1}}=-\frac{1}{2}{E}_{x\sim {p}_{g}}\left[log\left(D\left(x\right)\right)\right]-\frac{1}{2}{\mathbb{E}}_{x\sim {p}_{g}}\left[log\left(D\left(x,c\right)\right)\right]$$

where $x$ is a generated latent vector sampled from the distribution ${p}_{g}$. The first half of the equation is the semantic realism adversarial loss that distinguishes whether the latent feature is real or fake. The second half is the molecule–target pair interaction adversarial loss, which is used to determine whether molecules and protein targets could interact with each other.

A supervised contrastive loss was further proposed to better train generators. Traditional conditional generation models tend to focus on whether a correspondence exists between the generator and the result, and they do not consider the relationship with other conditions. A notable detail is that different protein targets correspond to molecules with different distributions in chemical space. The molecules generated from different targets are expected to better capture the characteristics of the original molecule. A resurgence of CL applied to self-supervised representation learning has occurred, achieving state-of-the-art performance in the unsupervised training of deep image models. Some works have also extended self-supervised batch comparison methods to a fully supervised setting, allowing to make effective use of labelling information. As shown in the bottom of bottom of Fig. 2a, clusters of points belonging to the same category were clustered together in the embedding space while separating clusters of samples from different categories. Inspired by [28], different protein targets could be seen as different labeling information. The following formula was utilized to calculate the supervised contrastive loss:

$${\mathcal{L}}_{{G}_{SCL}}={\sum }_{i=1}^{N} -\frac{1}{{N}_{{y}_{i}}-1}{\sum }_{j=1}^{N} {1}_{i\ne j}{1}_{{y}_{i}={y}_{j}}\mathrm{log}\frac{\mathrm{exp}\left(\Phi \left({x}_{i}\right)\cdot\Phi \left({x}_{j}\right)/\tau \right)}{{\sum }_{k=1}^{N} {1}_{i\ne k}\mathrm{exp}\left(\Phi \left({x}_{i}\right)\cdot\Phi \left({x}_{k}\right)/\tau \right)}$$

where $N$ is the batch size; $x$ is the latent vector of molecules; $\Phi \left(\cdot \right)$ denotes an encoder that outputs the L2 normalized final encoder hidden layer, and $y$ is the corresponding target label for each molecule.

The final objective function of the generator is defined as follows:

$${{\mathcal{L}}_{G}={\mathcal{L}}_{{G}_{1}}+\lambda \mathcal{L}}_{SCL}$$

where $\lambda$ is a loss weight to handle the importance of adversarial loss and the supervised contrastive loss.

The discriminator D was trained alternately to distinguish the inputs generated by the generator G or real data. Similar to the generator, the objective function of the discriminators includes semantic realism adversarial loss and molecule–target pair interaction adversarial loss, which is defined as follows:

$${\mathcal{L}}_{D} = - {\mathbb{E}}_{{x \sim P_{r} }} \left[ {D\left( x \right)} \right] + {\mathbb{E}}_{{x \sim P_{g} }} \left[ {D\left( x \right)} \right] - {\mathbb{E}}_{{x \sim P_{r} }} \left[ {D\left( {x,c} \right)} \right] + {\mathbb{E}}_{{x \sim P_{g} }} \left[ {D\left( {x,c} \right)} \right] + \lambda {\mathbb{E}}_{{x \sim {\mathcal{P}}_{{\hat{x}}} }} \left[ {\nabla _{x} \left( {D\left( x \right) + D\left( {x,c} \right)} \right)_{p} - 1} \right]^{2}$$

where ${P}_{r}$ is the distribution of real data. The final part of the equation is the gradient penalty mentioned in [32].

## Metrics

The effectiveness of our model was evaluated by the following approaches. First, the affinity and docking scores [37] were calculated to measure the binding capability of molecule and proteins. DeepPurpose [38] is a DL model-based molecular modeling and prediction toolkit that takes into an array of drug’s SMILES strings, an array of target protein’s amino acid sequence, and an array of label. It could either be binary 0/1 indicating interaction outcome or a real number indicating affinity value. We re-trained a model by merging our own dataset with BindingDB [39] and used the model to predict the generated data. For the Drug Encodings and Target Encodings, we selected Convolutional Neural Network on both SMILES and Targets. By utilizing the pre-trained model of DeepPurpose, the affinity scores of compounds and proteins could be obtained. Typically, the higher the score is, the stronger the affinity.

Molecular docking is a process in which two or more molecules recognize each other through ensemble matching and energy matching. Docking score, which could be calculated by molecular docking, has shown an important value in drug design. The glide in the Schrodinger suite [40], which is a drug discovery software platform that integrates visualization, modeling, simulations, and methodology development, was used to calculate the docking score. This score can be employed to rank the candidate poses as the sum of the electrostatic and van der Waals energies, and measure how well the generated molecules fit the binding site. For the docking score, a lower value means better.

Second, an assessment platform called MOSES [41], which proposes a set of metrics, was applied to evaluate the quality of generative models. The proposed metrics can detect common issues, such as overfitting, imbalance of frequent structures, and mode collapse. This method randomly chooses 30,000 generated molecules and applies this set to evaluate each of the following metrics: valid, unique, novelty, and property distribution**.**

Fraction of valid (Valid) and unique (Unique@k) molecules means validity and uniqueness of the generated SMILES strings, respectively.

Novelty is the fraction of the generated molecules that are not present in the training set. A high value of novelty indicates not overfitting.

Property distribution provides a visual representation of the properties of the resulting molecular structure.

- Molecular weight (MW): the sum of atomic weights in a molecule. By plotting the molecular weight histogram, whether the molecules generated are heavy or light could be determined.
- Lipophilicity (LogP): the lipophilicity of a compound is usually estimated by the LogP of the octanol/water partition, which is closely related to solubility, permeability, metabolism, and toxicity.
- Natural product-likeness (NP-likeness): a good source of validated substructures for the design of novel bioactive molecules.
- Synthetic accessibility score (SAscore): a heuristic estimate of how hard (10) or how easy (1) it is to synthesize a given molecule. SA score is based on a combination of the molecule’s fragments contributions.
- Quantitative estimation of drug-likeness (QED): a [0,1] value estimating how likely a molecule is a viable candidate for a drug. QED is meant to capture the abstract notion of aesthetics in medicinal chemistry.

## Results

### DeepTarget framework

DeepTarget consists of three submodules, namely, Amino Acid Sequence Embedding (AASE), Structural Feature Inference (SFI), and Molecule Generation (MG), corresponding to target sequence embedding module, molecule latent feature inference module, and molecule generation module, respectively. In AASE, it takes the sequence of the protein as input and embeds it to obtain features that represent the protein target. SFI generated molecule feature by applying the architectures of GANs, which exerts the idea of adversarial to force the network to generate results that interact with the protein. For generative models, the sampling distribution of chemical space generated under the same protein as conditions is often highly variable. Therefore, CL is added to the model to further optimize the chemical space and better capture the characteristic similarity of molecules under similar targets. In the end, the molecule representation is decoded to a specific molecule.

### Affinity prediction and molecular docking of the generated molecules to the target

**Fig. 3:**
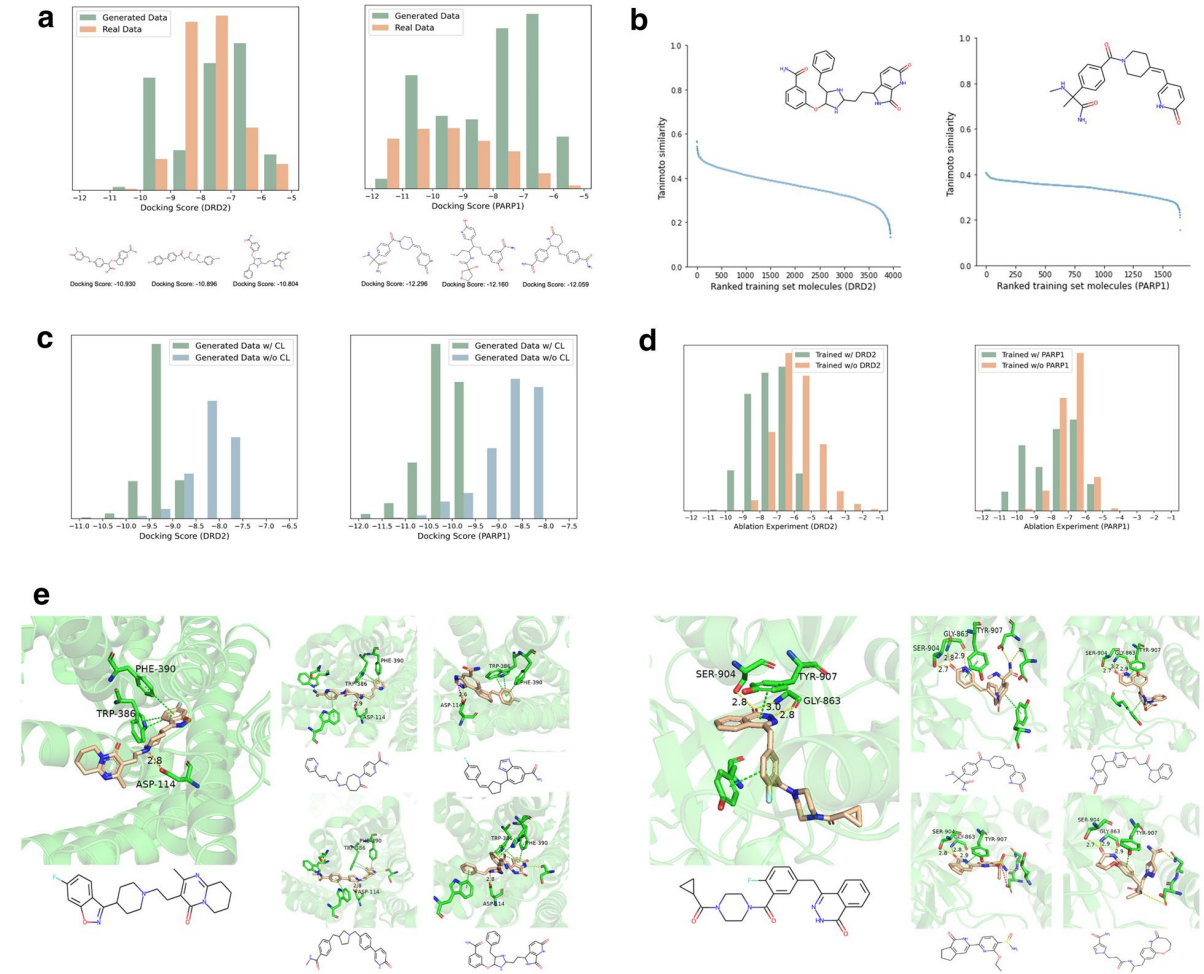
**a** The docking score result and some samples with top score of DRD2 (left) and PARP1 (right). Green is known molecules, yellow is generated. The Lower docking score indicates higher binding affinity. **b** Tanimoto similarity between generated molecule and each molecule in the training dataset. **c** The difference of top 1000 molecules’ Docking Score distribution when contrastive learning is or isn’t used. The left is from DRD2 and the right is from PARP1. **d** The docking score result of Zero-shot generation to unseen targets. **e** Generated molecules with top binding affinity and the reference molecule for representative binding sites. The known inhibitors (the big image) and the generated molecules (the small four images) of DRD2 (left) and PARP1 (right)

Whether the generated molecules could bind the target protein is the most important purpose. Two binding sites (PDB ID: 6cm4 and PDB ID: 7kk4) coming from DRD2 (Uniprot ID: P14416) [42] and PARP1(Uniprot ID: P09874) [43] were selected to calculate the docking score, QED, and SA for each of the generated molecules. Figure 3a presents the histogram of the docking score through Schrodinger and some generated samples with high score. Tables 1 and 2 show the mean values over all generated molecules. The two examples in the above results show some top affinity molecules could achieve docking, QED, and SA scores comparable to or even higher than those of the reference molecule. The available inhibitors of the target proteins were compared with the generated molecules to understand the results further intuitively. DRD2 and PARP1 were used as examples. For the DRD2 protein binding site, the three active site residues PHE-390, TRP-386, and Asp-114 are known to interact with various highly selective DRD2 inhibitors, which are also found to interact with generated novelty molecules, reported in literature. The left of Fig. 3e illustrates the interactions between the original active molecule and four representative generated molecules with these key active site residues. In the same manner, for the PARP1 protein binding site, the new generated molecules could interact with active site residues SER-904, TYR-907, and GLY-863 (shown in the right of Fig. 3e). These results indicated that the generated molecules could interact with the binding site, same as known inhibitors. Thus, the model could potentially learn information about the interaction of the binding site with the molecule, without informing any other information (only the protein sequence).

**Table 1:** Mean values of three metrics (docking score, QED, and SAscore) on generation quality of DRD2

Metric(Mean Value)	This Work	Real
Docking Score (kcal/mol, ↓)	− 8.8904	− 7.7532
QED (↑)	0.6402	0.6016
SAscore (↑)	3.0755	2.6886

(↑) means higher is better, and (↓) means lower is better

**Table 2:** Mean values of three metrics (docking score, QED, and SAscore) on generation quality of Parp1

Metric(Mean Value)	This Work	Real
Docking Score (kcal/mol, ↓)	− 9.7163	− 9.8276
QED (↑)	0.6776	0.6477
SAscore (↑)	2.8217	3.1649

(↑) means higher is better, and (↓) means lower is better

**Fig. 4:**
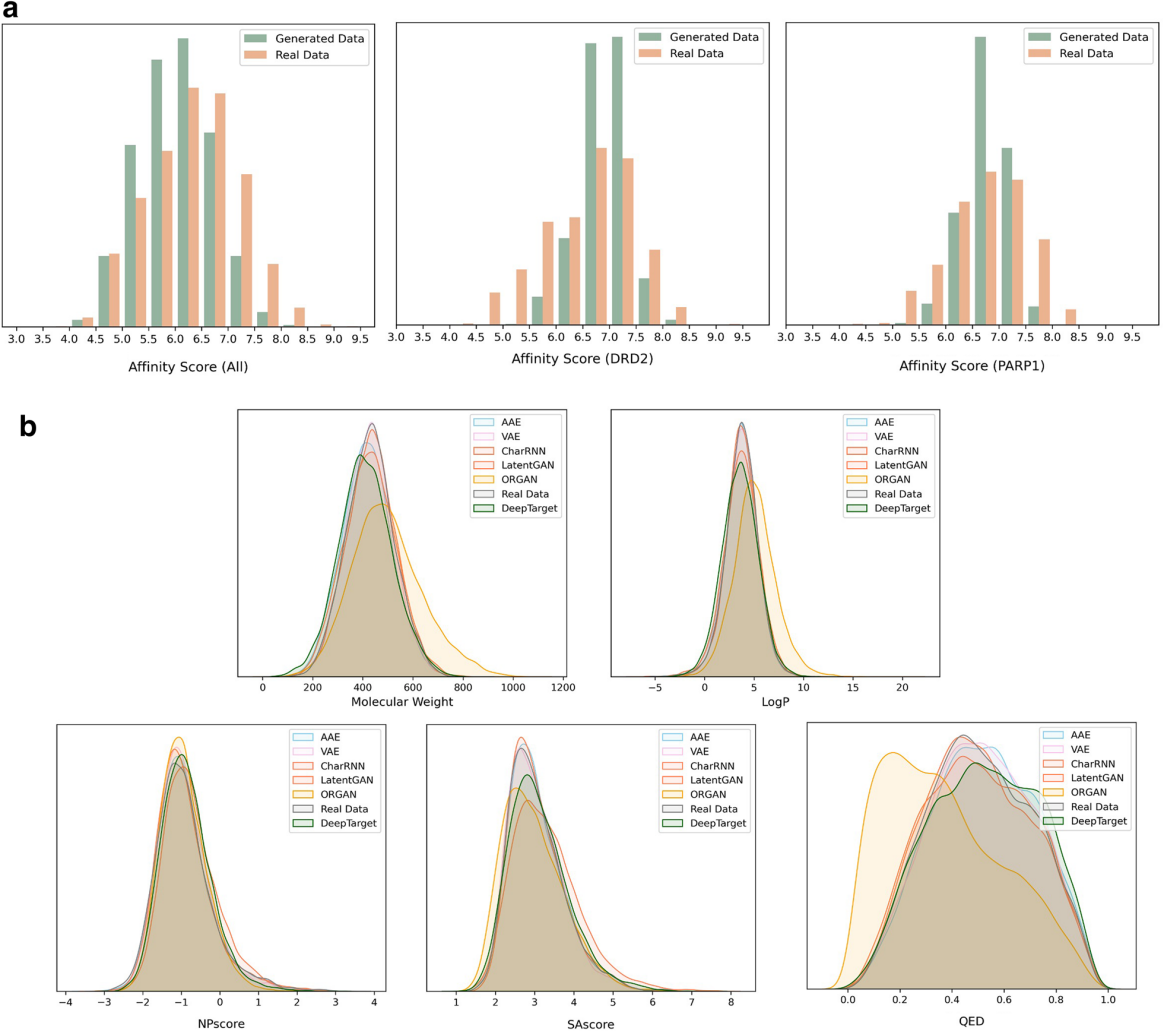
**a** Drug-Protein affinity distributions for randomly selected drug-protein pairs in the generated molecules and for drug-protein pairs in the original dataset (the left). Drug-Protein affinity of generating molecules and known active molecules of DRD2 and Parp1 (the two on the right). **b** Distributions of property values for the generated compounds. Properties include, Lipophilicity (LogP), Molecular Weight (MW), Natural Products-likeness (NP-likeness), synthetic accessibility Score (SAscore) and Quantitative Estimation of Drug-likeness (QED)

Drug–target affinity prediction is a commonly used method to conduct virtual screening. By applying DeepPurpose [38], all the affinity scores about the drug-target pairs were calculated in the present study. Afterwards, a number of generated molecules was randomly selected, and the scores were calculated. Figure 4a illustrates the distribution of real data and generated data. Clearly, the overall distribution was generally consistent. In detail, the two common binding targets DRD2 and PARP1 were still selected. The results of randomly chosen samples (Fig. 4a) show that in general, our model is able to discover molecules with similar binding affinity to targets.

### Augmentation of docking score by CL

The top 1000 molecules of the docking scores were selected from the results of the models with and without the inclusion of individual comparative learning for comparison. Figure 3c shows the histogram of DRD2 (left) and PARP1 (right). Better docking results could be obtained with the addition of CL.

### The novelty of the generated molecules

As mentioned above, the LBMG has the problem that hard to generate molecules with novel structures. In addition, a representative generated molecule shown in Fig. 3b from DRD2 and PARP1 was chosen to calculate the Tanimoto similarity with the training data. The similarity between this molecule and the training set was not high, both being below 0.6 (DRD2) and 0.4 (PARP1), as the similar curves indicated. It means that we can generate new molecules that could interact with their targets and also have novelty structures.

### Quality of generative models and property distribution of generated molecules

As mentioned in MOSES [41], in Table 3, the Unique@K for the first K = 1,000 and K = 10,000 valid molecules in the generated set was computed. The proposed condition-based model performed similarly to other generative models with a high percentage of novelty and unique SMILES strings. However, in the case of valid molecules, it showed a slightly inferior performance but higher than other GAN models. The reason is possibly because GANs are harder to train than other models. However, generating completely valid molecules is not our ultimate goal; few molecules that do not match the rules could be screened by a simple algorithm. Compared with works that focus only on the effective molecules, this study focused on the binding power of the generated molecules with the target protein.

**Table 3:** Valid, unique, and novelty values of compounds generated in five generation models

Model	Valid	Unique@1 k	Unique@10 k	Novelty
AAE	0.8282	0.994	0.9662	0.9972
VAE	0.8720	0.992	0.9564	0.9966
CharRNN	0.9842	0.998	0.9563	0.9953
ORGAN	0.6481	0.998	0.9793	0.9990
LatentGAN	0.7184	1.0	0.9994	0.9987
DeepTarget	0.8083	0.997	0.9926	0.9989

In addition, a total of 30,000 corresponding generated molecules were randomly selected to calculate QED, LogP, SAscore, NPscore, and MW. The property distribution (Fig. 4b) of all molecules was analyzed in comparison with other models. The distribution of property values produced by the proposed method was similar to that produced by other methods.

### Zero-shot generation to unseen targets information

Zero-shot generation (no known ligand information in training). Zero-shot ligand generation is very important for real world applications since we cannot always find active off-the-shelf ligands. In this section, we erased all DRD2 and PARP1 data from training set and retrained DeepTarget. After training, DRD2 and PARP1 were input to DeepTarget to generate molecules and 10,000 generated molecules were randomly chosen from each protein for molecule docking. As shown in Fig. 3d, DeepTarget could synthesize molecules whose docking scores were below -6 in most cases for DRD2. Potentially promising ligands could still be generated despite a slightly lower performance. In addition, we got the similar results for PARP1.

### Comparison with model based on machine translation mechanism

In this part, we made some comparisons for DeepTarget with a protein-specific de novo drug generation methods based on sequence-to-sequence model (in the following, we will refer to this as Seq2Seq). The training set for DeepTarget were re-trained in Seq2Seq and the two proteins (DRD2 and Parp1) were also applied to take the case study comparison.

Because of the complexity and time-consuming of the molecular docking process, we only generated 1000 molecules to calculate the valid, unique and novelty separately and randomly selected 500 to dock for each protein. In [44], it used beam search to sample relative data (one-per-one mode or ten-per-one mode). Beam search is sampled according to the magnitude of the probability, which could obtain the results with the higher combination probability (especially in one-per-one mode). However, it also has obvious drawbacks, i.e., as beam size increases, memory usage increases, the valid of generation decreases. The same conclusions are also shown in [44] with ten-per-one mode having bad value than one per one mode. In Additional file 1: Table S1, compared to DeepTarget, it can be observed that Seq2Seq has lower valid than the former and got close to unique and novelty. Next, datasets that erased DRD2 and PARP1 were also trained by Seq2Seq. For each protein, 500 generated molecules were selected to take affinity score prediction and molecule docking. The distributions for the predicted affinity score are illustrated in Additional file 1: Figure S1. Meanwhile, their means and standard deviation are calculated in Additional file 1: Table S2. Clearly, the molecules generated by DeepTarget show slightly higher values than Seq2Seq, while they also could get similarity distribution. As for the docking score, in Additional file 1: Table S3 and Additional file 1: Figure S2, DeepTarget and Seq2Seq could get close distributions on Parp1, while the better value is obtained from DeepTarget. In terms of DRD2, both in terms of mean and distribution charts, DeepTarget has the advantage of being visible in the flesh.

The comparison of these two methods indicated that both are capable of generating ligand molecules against protein sequences and our model has a greater advantage. On the one hand, in the generated results for untrained protein sequences, our model generates molecules with better interactions. On the other hand, drug design need screening suitable molecules from a large number of candidates, which means not simply generating small batches of molecules. In terms of Seq2Seq, the larger the size of the beam search, the more memory rises sharply and various performance degradation [45], which is not suitable for large data generation. Instead, this is not the case for generative models based on probabilistic sampling and it could generate different data in large batches. In conclusion, our model has better applicability.

## Discussion

Most DL methods normally involve a known activity library of molecules or bind pockets of a particular protein to produce ligand. In other words, these methods require some prior information about the compounds that are active or complex self-information of the target protein. In general, the methods based on known compounds could not generate novel results. Even some proteins also do not have sufficient known molecules. In addition, the determination of the 3D structure of a protein is not an easy task, and it is quite costly. Conversely, the proposed method does not require in-depth knowledge of any kind of chemical descriptor of the active ligand or molecule nor does rely on information about the 3D structure of the protein. Consequently, the use of amino acid sequences as input to discover molecules could considerably simplify the initial phase of drug discovery.

Our study was to generate SMILES molecules simply on the basis of amino acid sequences. The method benefits from the recent progress in the image or text generation field, where this architecture shows state-of-the-art results. First, GAN is an unsupervised learning model whose most important feature is that it provides a method of adversarial training for deep networks. Such method helps better learn the distribution and relationship between data. Second, the products of GANs have greater diversity than machine translation. In addition, the introduction of CL greatly enhances the quality of the generated results, thus better closing the chemical space features of the same protein target. In addition, our methods have rapid process to generate a series of candidate molecules. It has been tested that our model could generate novel molecules at a rate of about 980/s on a single 40G NVIDIA A100 (Ubuntu 18.04, AMD EPYC 7742 64-Core Processor).

The above results show that the idea of generating molecules with interrelationships directly from protein target sequences has a certain plausibility. For the generated molecules, the generative effect of the model and the properties of the molecules are similar to the capabilities of the other models. For the target protein, the model could generate molecules that are similar to known active molecules and obtain novel new molecules. Importantly, the affinity scores of the generated molecules are not only related to the distribution of the known active molecules, but their interaction ability could be demonstrated by molecular docking.

## Supplementary Information

**Additional file 1:** Figures and Tables of the comparison method.

## Data Availability

Code and data are available at an opensource GitHub repository at: https://github.com/viko-3/TargetGAN.
