# Supplementary material for: Deep generative model for drug design from protein target sequence
Source: J Cheminform. 2023 Mar 28;15:38. doi: 10.1186/s13321-023-00702-2 (PMC10052801; doi:10.1186/s13321-023-00702-2)
Supplement: Supplementary file 1 — Additional file 1: Figures and Tables of the comparison method. [file 13321_2023_702_MOESM1_ESM.docx]

**Additional file 1: Table S1** Valid, unique, and novelty values for Seq2Seq2 methods and our methods (DeepTarget).

| Proteins | Model | Valid | Unique | Novelty |
| --- | --- | --- | --- | --- |
| DRD2 | Seq2Seq | 0.70 | 0.995 | 0.966 |
|  | DeepTarget | 0.81 | 0.999 | 0.999 |
| Parp1 | Seq2Seq | 0.72 | 0.999 | 0.999 |
|  | DeepTarget | 0.82 | 0.999 | 0.999 |

**Additional file 2: Table S2** Mean and standard deviation values of affinity score for DeepTarget and SeqSeq.

| Affinity Score | | **DeepTarget**  **(DRD2)** | | **Seq2Seq**  **(DRD2)** | | **DeepTarget**  **(Parp1)** | **Seq2Seq**  **(Parp1)** |
| --- | --- | --- | --- | --- | --- | --- | --- |
| Mean | 7.728 | | 7.276 | | 7.983 | | 7.699 |
| Std | 0.404 | | 0.241 | | 0.315 | | 0.233 |

**Additional file 3: Table S3** Mean and standard deviation values of docking score for DeepTarget and SeqSeq.

| Docking Score | | **DeepTarget**  **(DRD2)** | | **Seq2Seq**  **(DRD2)** | | **DeepTarget**  **(Parp1)** | **Seq2Seq**  **(Parp1)** |
| --- | --- | --- | --- | --- | --- | --- | --- |
| Mean | -6.719 | | -5.523 | | -6.792 | | -6.727 |
| Std | 0.894 | | 1.139 | | 0.826 | | 0.941 |


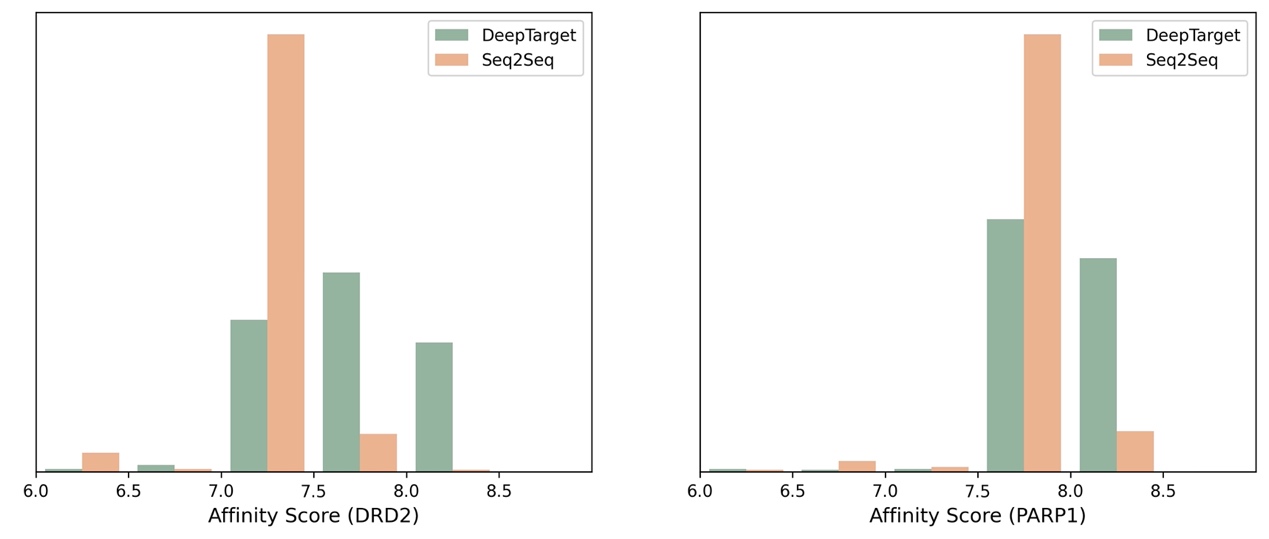


Additional file 1: Figure S1 Drug-Protein affinity score of generating molecules and known active molecules of DRD2 (left) and Parp1 (right).


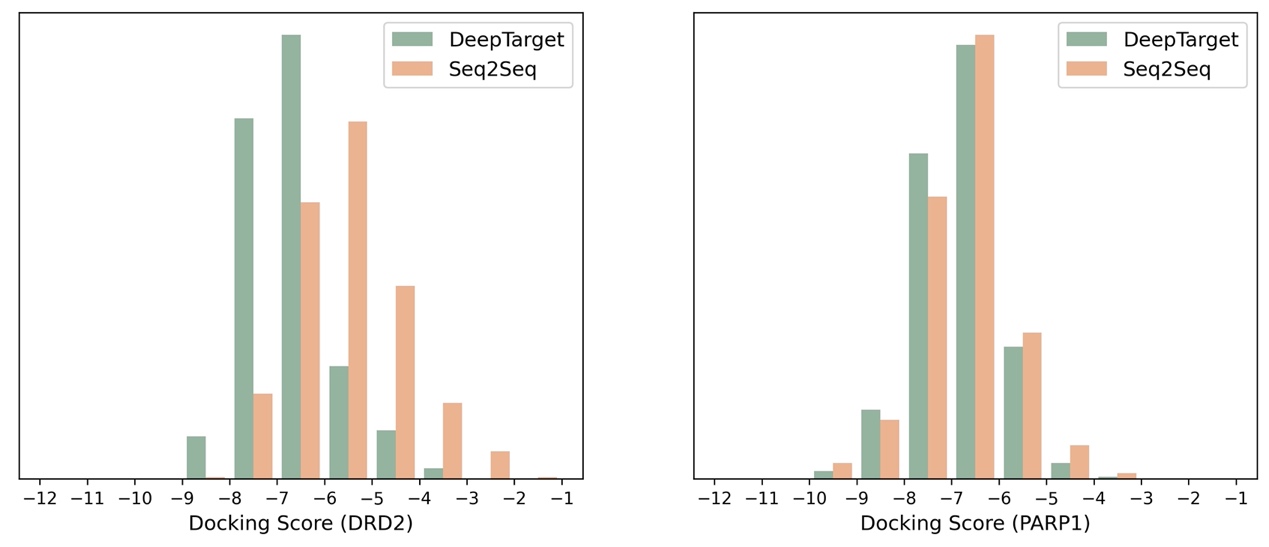


Additional file 1: Figure S2 Drug-Protein docking score of generating molecules and known active molecules of DRD2 (left) and Parp1 (right).

**Reference:**

1. Grechishnikova, D., *Transformer neural network for protein-specific de novo drug generation as a machine translation problem.* Scientific reports, 2021. **11**(1): p. 1-13.
